# Melt‐Encoded‐Tags for Expanded Optical Readout in Digital PCR (METEOR‐dPCR) Enables Highly Multiplexed Quantitative Gene Panel Profiling

**DOI:** 10.1002/advs.202301630

**Published:** 2023-07-23

**Authors:** Dong Dong Liu, Daniel Muliaditan, Ramya Viswanathan, Xu Cui, Lih Feng Cheow

**Affiliations:** ^1^ Institute for Health Innovation and Technology National University of Singapore Singapore 117599 Singapore; ^2^ Department of Biomedical Engineering Faculty of Engineering National University of Singapore Singapore 117583 Singapore; ^3^ Genome institute of Singapore Agency for Science Technology and Research Singapore 138672 Singapore

**Keywords:** copy number analysis, digital PCR, melt‐curve analysis, multiplexing

## Abstract

Digital PCR (dPCR) is an important tool for precise nucleic acid quantification in clinical setting, but the limited multiplexing capability restricts its applications for quantitative gene panel profiling. Here, this work describes melt‐encoded‐tags for expanded optical readout in digital PCR (METEOR‐dPCR), a simple two‐step assay that enables simultaneous quantification of a large panel of arbitrary genes in a dPCR platform. Target genes are quantitatively converted into DNA tags with unique melting temperatures through a ligation approach. These tags are then counted and distinguished by their melt‐curve profiles on a dPCR platform. A multiplexing capacity of *M*^*N*, where *M* is the number of resolvable melting temperature and *N* is the number of fluorescence channel, can be achieved. This work validates METEOR‐dPCR with simultaneous DNA copy number profiling of 60 targets using dPCR in cancer cells, and demonstrates its sensitivity for estimating tumor fraction in mixed tumor and normal DNA samples. The rapid, quantitative, and highly multiplexed METEOR‐dPCR assay will have wide appeal for many clinical applications.

## Graphical abstract

The affinities of fluorescence probes hybridized to DNA barcodes (BCs) are governed by their degree of complementarity, and can be tuned to produce a wide range of thermal melting profiles. Using this principle, melt‐encoded‐tags for expanded optical readout in digital PCR (METEOR‐dPCR) associates unique DNA BCs to specific targets to enable highly multiplexed quantitation of large gene panels.

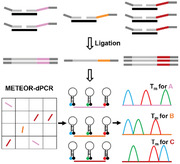

## 1 Introduction

Advances in next generation sequencing (NGS) have fundamentally transformed the way we approach healthcare, in the aspect of early disease detection, patient stratification and treatment monitoring.^[^ ^1^ ^]^ For example, noninvasive cancer detection can be achieved by sequencing the cell‐free DNA (cfDNA) from blood.^[^ ^2^ ^]^ As genomic copy‐number variations (CNV) are common in cancer,^[^ ^3^ ^]^ the alterations in sequencing coverage depth across different regions in cfDNA would be a specific feature of cancer patients.^[^ ^4^ ^]^ Noninvasive monitoring of cancer patient response to treatment can also be achieved by monitoring the degree of CNV in cfDNA over time.^[^ ^5^ ^]^ The key features of NGS that enabled these applications are its superior quantitative ability and multiplexing capacity. However, this comes at a cost of a complex and expensive workflow and typical weeks of processing time from a central facility—sacrificing precious time for patient treatment.

Digital PCR (dPCR) is a promising new technology that is simple to perform (hours for results) and yields absolute quantitative measurements that surpasses even NGS.^[^ ^6^ ^]^ In dPCR, target molecules are individually partitioned in a massive array of nanoliter chamber or droplet and amplified. Simple enumeration of the partitions that contains PCR products will give accurate measurement of absolute numbers of target molecules.^[^ ^7^ ^]^ Due to its sensitivity, dPCR is able to quantify small changes in copy numbers and is insensitive to amplification biases commonly encountered in bulk assays.^[^ ^8^ ^]^ However, the use of dPCR for gene copy number detection has been limited to a small number of targets. The lack of multiplexing capability in dPCR has been a deterrent to it becoming a replacement of NGS in these important clinical applications that require measurements of multiple chromosomes and loci simultaneously.

To date, the multiplexing capability of dPCR is fundamentally limited by two factors. Firstly, it is notoriously challenging to design a multiplexed PCR assay for multitarget amplification. In practice, multiplexed PCR primers can be optimized for only a handful of targets, beyond that nonspecific amplification will invariably become a problem.^[^ ^9^ ^]^ Secondly, multiplexing in dPCR is limited by the number of fluorescence channels in the instrument. Although to some extent the multiplexing capacity can be extended by using melt characteristics,^[^ ^10^ ^]^ kinetic (amplification curve) analysis,^[^ ^11^ ^]^ incorporating probes with different fluorescence intensities,^[^ ^12^ ^]^ or even applying deep learning to distinguish probes with overlapping fluorescence spectrum,^[^ ^13^ ^]^ the need for large number of fluorescence probes increase the cost, complexity and potential for nonspecific signals. Due to these challenges, a scalable and practical multitarget assay based on dPCR has not been realized.

Here, we present a novel methodology for massively scaling up the multiplexing capability of dPCR for gene copy number detection. Different from conventional approaches where sample DNA is directly amplified, our approach first generates intermediate Melt‐Encoded‐Tags (MET) based on the abundance of each DNA target in the sample. While all MET include a set of universal primer binding sites, tags corresponding to each target are distinguished by the sequences in their barcode (BC) region. The different BC sequences are designed to bind to a set of fluorescent sloppy molecular beacon (SMB) probes with different affinity, to yield unique thermal dissociation profiles. Multiplexed gene copy number profiling is performed by enumerating the METs in a dPCR platform, where the identities of the METs are determined by melt‐curve analysis upon hybridization with the SMB probes. We refer to this novel method as Melt‐Encoded‐Tags for Expanded Optical Readout in digital PCR (METEOR‐dPCR), to reflect the unique melt‐curve profiling strategy enabling significant expansion of multiplexing capability for dPCR‐based multigene detection.

We first showed that Tm signatures enabled expansion of the multiplexing capacity in each fluorescence channel. Representing each gene with one fluorophore at one of three distinct Tm values, we demonstrate accurate copy number profiling of nine target genes (3 + 3 + 3) in a 3‐channel dPCR platform. Next, we further extended the multiplexing capacity by representing each gene target simultaneously with three fluorophores at varying Tm values. An unprecedented multiplexing capacity of 60‐plex (4 × 5 × 3) for simultaneously assaying 20 genes in three samples in a single assay is achieved. We demonstrate accurate profiling of copy number changes in two multiple myeloma cell lines associated with clinical risk and prognosis. Furthermore, we validated copy number profile changes across multiple gene during disease progression of patient‐derived head and neck cancer cell lines. Finally, we showed that the sensitivity of METEOR‐dPCR allowed us to detect copy number changes of cancer cells in an excess of normal cells, laying the foundation for potential future applications in liquid biopsy.

## 2 Results

### 2.1 Principles of METEOR‐dPCR

To realize a robust dPCR system that can perform highly multiplexed gene panel profiling, we considered a few important criteria. Firstly, the assay should be scalable and easily customized for specific target genes, without the need to perform extensive optimization (e.g., primer and probe design, reaction conditions) after any modifications. Secondly, fluorescent probes are a major cost in nucleic acid assays, it is preferable to avoid the need for new fluorescent probes for every gene to be detected. In an ideal situation, a set of universal probes can be used for any gene. Lastly, gene panel assays should be able to detect 20–100 targets. Single gene copy number analysis is well served by conventional dPCR assays while genome‐wide copy number profiling is better suited with NGS technologies. There is an unmet need for rapid and cost effective gene copy number assay for intermediate number of genes (e.g., covering CNV hotspots in cancer, or chromosomes implicated in fetal genetic aneuploidy) in diagnostics and screening applications.

Multiplexed PCR is the most direct approach for multigene detection,^[^ ^16^ ^]^ however they require extensive optimization to ensure specificity. Moreover, the fluorescence probes required for multiplexed PCR scales linearly with the number of targets. As such, multiplexed PCR detection for >10 targets is exceedingly challenging. A more scalable solution for multiplexed gene copy number profiling could be by generating intermediate “tag” molecules according to the abundance of respective targets in the sample. Several notable examples of tag‐based methods for gene copy number profiling include the hybridization‐based MAPH^[^ ^17^ ^]^ and Nanostring assays,^[^ ^18^ ^]^ as well as the ligation‐based MLPA^[^ ^19^ ^]^ and digital MLPA assays.^[^ ^20^ ^]^ A clear advantage of the tag‐based approach is the flexibility to incorporate additional functions into the tag to facilitate downstream detection. For example, the tags in Nanostring assay^[^ ^18^ ^]^ are functionalized with fluorophores for optical detection, tags in MAPH and MLPA assays are appended with variable‐length stuffer sequences to allow them to be separated in capillary electrophoresis,^[^ ^19^ ^]^ and tags in digital MLPA assay contain specific DNA sequences that allowed them to be identified with next generation sequencing.^[^ ^20^ ^]^ Interestingly, despite the clear advantage of dPCR platforms for nucleic acid quantification, they have seldom been explored for tag‐based quantifications, due to the current lack of multiplexing capability in these platforms.

We have recently shown that SMB ^[^ ^21^ ^]^ melt‐curve analysis in dPCR can provide unprecedented ability to discriminate and identify polymorphic sequences.^[^ ^14^ ^]^ These mismatch‐tolerant fluorescent probes bind to the polymorphic bacteria 16S rDNA with variable affinity due to different base mismatches, and can be unequivocally distinguished using multicolor melt‐curve analysis. Using this approach, we demonstrate that quantitative measurements of up to 16 different bacteria species in various mixtures are possible. Currently this approach is used to identify polymorphic sequences of the same gene, and is restricted by the natural sequence variations. In this work, we show that SMB melt‐curve analysis performed on BC sequences in tag molecules provides a novel method for highly multiplexed copy number analysis of arbitrary targets in dPCR.

**Figure 1:**
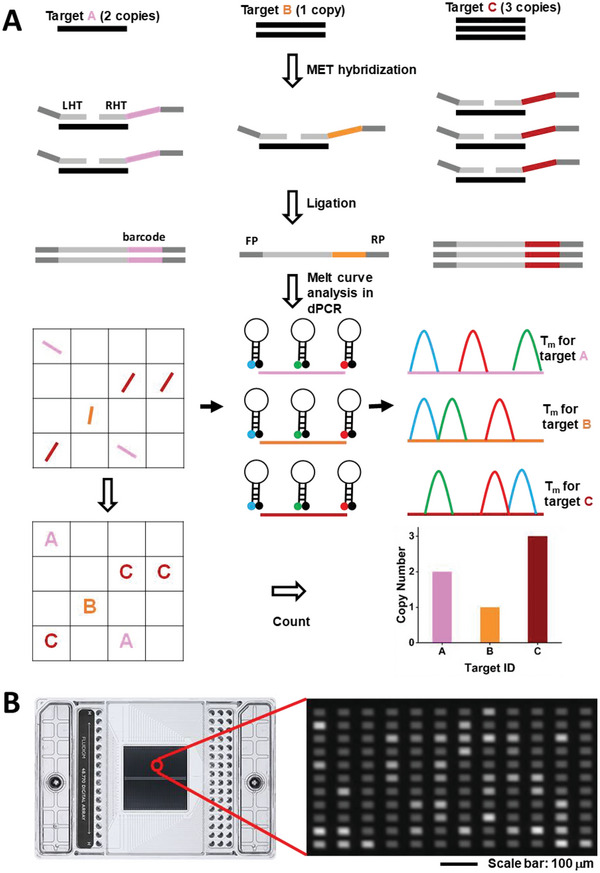
Schematic workflow of the Melt‐Encoded‐Tags for Expanded Optical Readout in digital PCR (METEOR‐dPCR) assay. A) Illustrating the use of METEOR‐dPCR to profile the copy numbers of three genes (A–C). Left half tags (LHT) and right‐half‐tags (RHT) containing barcodes (BCs) selectively hybridize with the target genes. Adjacent LHT and RHT are ligated to form Melt‐Encoded Tags (MET). The number of MET generated is proportional to the copy number of target genes. The METs are then partitioned into a dPCR chip and undergo asymmetric PCR with universal primers to generate single‐stranded copies. A panel (3) of fluorescently labeled sloppy‐molecular beacon (SMB) probes bind to the amplified METs with different mismatches and generate unique melt‐curve profiles during temperature ramping. By matching the melt‐curve profile in each partition with the expected Tm signature of each target MET, the copy number of multiple target genes can be simultaneously obtained. B) Digital PCR chip (dqPCR 37k IFC, Fluidigm) being used for this work and a magnified snapshot of florescence image captured during melt‐curve analysis.

A detailed schematic for METEOR‐dPCR is shown in **Figure** **1**. A ligation approach is adopted to generate METs corresponding to the abundance of each target. Each MET consists of a left half tag (LHT) and right half tag (RHT) that will bind next to each other to the target through their hybridization region. In addition, the LHT contains binding sequences for the universal forward primer and the RHT contains binding sequences for the universal reverse primer. Finally, a unique BC sequence is inserted between the hybridization region and the reverse primer sequences of the RHT. Firstly, the sample DNA is denatured and the half tags bind to the target region. Ligase is added to join the pairs of half‐tags that are brought together to close proximity by their cognate target. In this way, quantitative differences in the numbers of ligated METs occurs due to the copy number differences of the targets.

Identification of the METs is performed by examining the fluorescence signal of SMB probes as they hybridize to the BC sequences at different temperatures. The SMB probes are synthetic DNA sequences that form a stem‐loop structure, with a fluorophore and a quencher on each end of the sequence. When the SMB probes are in a stem loop structure, the fluorescence signal is effectively quenched. However, if the SMB probe binds to a complementary target sequence through its loop region, the fluorophore and quencher is separated and a fluorescence signal is obtained. The switch from an open (binding, high fluorescence) to closed (nonbinding, low fluorescence) state is dependent on the temperature and affinity of the binding sequence to the probe. If the binding sequence is perfectly complementary to the probe, the binding is strong and a high temperature is required to change the probe from an open to closed state. On the other hand, if there are multiple mismatches of the binding sequence to the probe, the binding is weak and the probe will favor a closed state. As such, the temperature at which the SMB probe transition from high fluorescence to low fluorescence is a signature of the underlying BC sequence. The final Tm of the BC and SMB probe depend on their respective sequences as well as the number and location of mismatches. The Tm of probe binding to DNA BC may be predicted from DNA binding thermodynamics ^[^ ^22^ ^]^ using freely available resources such as DINAMelt.^[^ ^15^ ^]^

The final piece of METEOR‐dPCR is the use of dPCR and melt‐curve analysis for multiplexed quantitative analysis of the METs. METs are isolated by partitioning the reaction across many nanoliter compartments in a dPCR chip. Massively parallel amplification of the METs are carried out in these compartments utilizing the common forward and reverse primers. Finally, melt‐curve analysis allows the precise identification of the METs in each compartment by their Tm signatures as described above. The ensemble of melt curves enables sensitive and quantitative copy number analysis of multiple genes simultaneously.

### 2.2 Multiplexed Qualitative Gene Detection with Bulk MET Melt‐Curve Analysis

**Figure 2:**
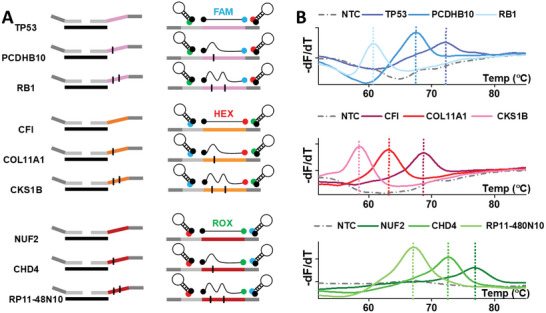
Basic strategy of encoding each target with a single barcode (BC). A) Schematic for generating Melt‐Encoded‐Tags (METs) with unique BCs corresponding to nine target genes. Three sloppy molecular beacon (SMB) probes (FAM, HEX, and ROX) are added to the reaction mix. Each MET contains a BC that can bind to one SMB probe (no binding to the other two SMB probes). Depending on the degree of mismatch (bulge in illustration) between MET BC and corresponding SMB probe, the melt‐curve profiles between different target genes can be distinguished. B) METs corresponding to each target gene were loaded into different wells, amplified, and hybridized with probes on a real‐time PCR platform (bulk assay). Melt‐curve analysis showed the detection of a distinct Tm for each of the MET. Nine target genes can be distinguished with three probes.

We first consider a simple Tm encoding scheme for METs. Each MET is encoded by two dimensions, its melting temperature and the type of fluorophore. The product of number of fluorescence channel (*N*) and the number of Tm in each channel (*M*) thus defines the overall multiplexing capacity (*N* × *M*). As a proof of concept, we designed METs for nine genes that frequently undergo copy number changes in cancer (Data S1, Supporting Information). Each of these METs contained a BC that will produce a distinct melting temperature when hybridized to one of three SMB probes (FAM, HEX, and ROX). The variable Tms of the BCs resulted from carefully introduced mismatched bases that altered their affinity to the respective probes (**Figure** **2**A). In this example, three distinct Tms in three fluorescence channels can be used to identify the nine METs, representing a significant expansion of multiplexing capability compared to conventional one‐color‐one‐target approach. When the pool of nine LHTs and nine RHTs are reacted in the presence of a single target gene, we observed a single peak with distinct Tm in the melt‐curve analysis after MET amplification with universal primers in a qPCR platform (Figure 2B). On the other hand, there was no peak observed in the no‐template‐control (NTC). These results demonstrate the excellent specificity of this approach.

**Figure 3:**
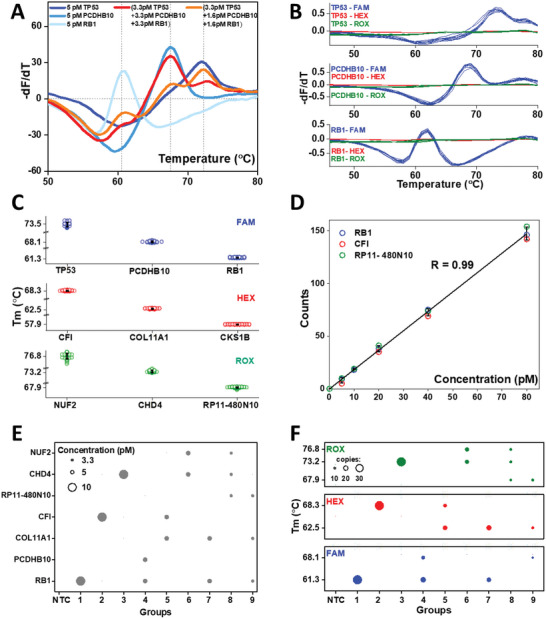
Melt‐Encoded‐Tags for Expanded Optical Readout in digital PCR (METEOR‐dPCR) enables simultaneous quantification of multiple targets. A) Real‐time PCR platform allowed detection of multiple Melt‐Encoded‐Tags (METs) (distinct peaks) in mixtures. However, there is no clear relationship between the melt‐curve profile and the relative quantities of METs. Partial merging of melt‐curve profile from different target METs affect their individual Tms and melt curves. Competition among different METs for binding to probes further complicates their interpretation. B) Single‐copy MET in nanoliter partitions within a dPCR chip can be amplified and hybridized to probes to produce consistent melt‐curve profiles (*n* = 10). Melt curves with the characteristic Tm of the MET were only observed in the fluorescence channel of the hybridizing probe. C) Reproducible and distinct Tms are obtained for each MET in their respective fluorescence channel in dPCR platform. Error bar shows standard deviation (*n* = 10). D) Linear relationship between the number of positive partitions (with expected Tm) in METEOR‐dPCR and input target gene concentrations. Error bar shows standard deviation (*n* = 3). E) Composition of test samples containing different mixtures of target gene concentrations to validate METEOR‐dPCR assay. F) Copy number of each MET measured on METEOR‐dPCR assay.

Due to the distinct Tms of the METs, multiple genes can be simultaneously detected in a qPCR platform. Three distinct melt‐curve peaks in each of the three fluorescence channels, corresponding to all nine METs, were detected when genomic DNA of JJN3 cells was tested (Figure S1, Supporting Information). However, there was no apparent quantitative relationship between the melt‐curve peaks and the abundance of specific target sequences. In a separate experiment where we tested genes with different input abundances, we found that there were large intrinsic variations between the peak heights of different METs. Moreover, the peak heights could change in a complex manner when multiple genes were simultaneously present in the samples (**Figure** **3**A, Figures S2 and S3, Supporting Information), potentially due to competition of different targets for the same probe. Thus, although bulk melt‐curve analysis could yield qualitative information about the presence of absence of specific genes in a sample, it is still inadequate to provide quantitative information of each target gene for copy number analysis.

### 2.3 Multiplexed Quantitative Gene Detection with METEOR‐dPCR

Our strategy to enable quantitative MET detection is to perform SMB melt‐curve analysis in a dPCR platform where individual MET is confined in separate partition. Tm signatures from many METs can be simultaneously obtained in a single dPCR chip, providing information about the identity and abundance of all the METS. Thus, the METEOR‐dPCR approach exploits digital melt‐curve analysis for highly multiplexed profiling of genomic copy number alterations (Figure 1a).

To demonstrate METEOR‐dPCR, we made use of the microfluidic‐based Fluidigm dPCR system (Figure 1b), as it has a large number of partitions (≈37 000) and is capable of performing melt‐curve analysis.^[^ ^23^ ^]^ METs prepared as described above are mixed with universal primers and SMB probes in a PCR mix and partitioned into nanoliter compartments in the dPCR chip. With limited dilution, each compartment most likely contains either one or zero copies of MET. Massively parallel asymmetric PCR is carried out in all compartments to generate clonal sequences of the starting template and finally melt‐curve analysis is performed to assess the binding of SMB probes to the BC sequences.

We first performed experiments to assess if robust melt curves can be obtained starting from single MET template in a dPCR platform. Nine types of METs with different BCs, corresponding to the nine genes described above are separately prepared, and METEOR‐dPCR was performed on limited dilutions of each MET in the dPCR platform. Figure 3B and Figure S4 (Supporting Information) (replicates from different chips) shows the raw melting curves in the first 10 positive compartments in digital melting PCR for three genes (TP53, PCDHB10, and RB1). We observed that the “digital” melting curve profiles for all METs are highly consistent, and a single peak allows robust determination of Tm. The Tm signatures of different METs are highly reproducible and also differ significantly allowing them to be identified unambiguously (Figure 3C, Table S1, Supporting Information). These results showed that asymmetric PCR from single molecules in nanoliter compartment is robust, and the melt‐curve analysis of SMB probes binding to its cognate target is highly specific on a widely accessible commercial dPCR platform.

The dPCR format can overcome the fundamental challenge of quantifying multiple targets by bulk melt‐curve analysis. By partitioning each MET in separate compartment, they can be precisely enumerated. To demonstrate this, we prepared samples containing different concentrations of synthetic gene sequences and quantified the METs produced with METEOR‐dPCR. The number of positive partitions with expected melt‐curve profiles in the dPCR chip are counted for each sample. Our results showed a linear relationship between the input sample concentration and the MET counts obtained from dPCR (Figure 3D, Table S2, Supporting Information). Importantly, similar linear correlation between the sample inputs and MET counts are observed for multiple targets, suggesting that MET counts is an accurate measure of target gene abundance in samples. Due to the proven ability of dPCR for very high‐resolution measurement of target quantity, it is ideal for detecting subtle copy number changes for disease diagnosis and monitoring

By combining the enhanced multiplexing capacity from melt‐curve analysis and accurate MET quantification with dPCR, we have an ideal platform for rapid multiplexed gene copy number quantification. To demonstrate simultaneous quantification of multiple genes, we prepared samples consisting of different amounts of synthetic sequences corresponding to our nine target genes (Figure 3E). We designed conditions with single target, multiple targets in the same fluorescence channel as well as multiple targets in different fluorescence channels to test the specificity of METEOR‐dPCR. The results from METEOR‐dPCR assay are shown in Figure 3F and Table S3 (Supporting Information) (results from three replicates), where the number of partitions with Tm values corresponding to each gene is summarized. We showed that different combinations and abundance of gene targets in each of these samples are accurately reflected by METEOR‐dPCR (Figure S5, Supporting Information). These results clearly demonstrate better specificity and accuracy of METEOR‐dPCR assay for DNA copy number measurement than a real‐time PCR platform (Figures S2 and S3, Supporting Information).

### 2.4 Further Expansion of Multiplexing Capacity with Multibarcode Tags

We have shown in the previous examples that a simple single‐BC encoding scheme can yield a multiplexing capacity of *N* × *M*, where *N* is the number of fluorescence channel and *M* is the distinct Tm in each channel. This is because the BC can hybridize with one SMB probe to yield distinct melt‐curve profile in one fluorescence channel. While this already represents a major improvement compared to existing one‐channel‐one‐target paradigm, a simple modification can in principle provide even further expansion to this strategy. This can be achieved by including multiple BCs (compound BCs) to each MET, where each BC can hybridize to a SMB probe with different fluorescence. In this way, the identity of each MET would be encoded by the Tm in ALL the fluorescence channels, and the multiplexing capacity is *M*^*N*.

**Figure 4:**
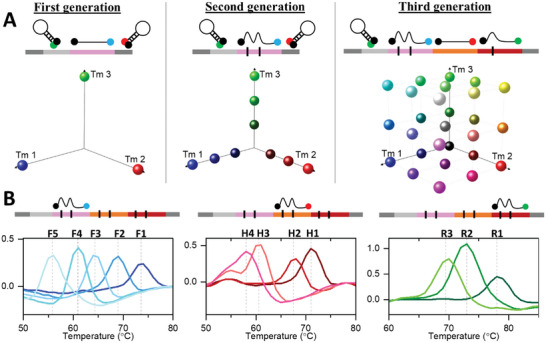
Advanced encoding scheme allows enhanced multiplexing in Melt‐Encoded‐Tags for Expanded Optical Readout in digital PCR (METEOR‐dPCR). A) Illustration of the multiplexing capacity in different encoding schemes. *N* represents the number of channels, *M* represents the distinct resolvable Tm in each channel. In the first‐generation scheme, each probe bind to only one MET and no melt‐curve analysis is performed. The multiplexing capacity is *N*. In the second‐generation scheme, each probe can bind to *M* METs with different mismatch patterns to generate *M* distinct Tm signatures. The multiplexing capacity is *N* × *M*. The third‐generation scheme extends the concept of the second‐generation scheme, but each MET can simultaneously bind to all *N* probes to generate Tm signatures in all fluorescence channels. The multiplexing capacity is *M*^*N*. The 3D plots below exemplifies the sets of valid Tm signatures in each scheme that can be used to distinguish targets. B) Clearly resolvable melt curves of five, four, and three distinct barcodes (BCs) in the FAM, HEX, and ROX channels. Inclusion of a BC from each channel in the METs would theoretically allow 60 unique compound Tm signatures to be obtained according to the third‐generation encoding scheme.

A conceptual schematic of the increasingly advanced encoding scheme leading to successive expansion in multiplexing is shown in **Figure** **4**A. In the first‐generation scheme, one‐channel‐one‐target paradigm, the target/BC is required to be perfectly complementary to the probes. Hence, the multiplexing capacity is exactly equal to the number of channels/probes (*N*). In the second‐generation scheme, a single BC is attached to each target and each BC can have different degrees of mismatch to its cognate probe to yield variable Tm (*M*). No Tms are obtained in the channels where the probes do not bind to the BC. The multiplexing capacity is *N* × *M* in the second‐generation scheme. Finally, in the third‐generation scheme, *N* BCs are attached to each target so that they participate in hybridization to ALL *N* probes. By virtue of the vast number of possible Tm combinations from each channel, this strategy has the highest multiplexing capacity of *M*^*N*. The capacity of various encoding scheme is also visualized in Figure 4A (*N* = 3, *M* = 3). The first‐, second‐, and third‐generation scheme can detect 3, 9, and 27 targets respectively, highlighting the potential for extensive multiplexing in dPCR platform with a novel barcoding strategy.

**Figure 5:**
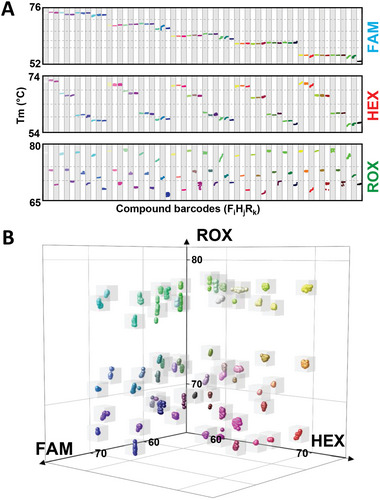
Robust identification of compound barcodes (BCs) based on multicolor Tm profiling. A) Distinct and reproducible Tm profiles of 60 synthetic compound BC sequences in dPCR chip. The compound sequence was generated from combinations of five BCs from FAM channel, four BCs from HEX channel, and three BCs from ROX channel. Hundred measurements per compound BC shown B) 3D scatter plot of the Tm values from 60 synthetic compound BC sequences in METEOR‐dPCR (40 measurements per sequence shown). Gray cubes are 1 °C × 1 °C × 1 °C regions centered on the median Tm of each compound BC.

To demonstrate the degree of multiplexing that can be achieved with third‐generation encoding scheme, we designed five separate BCs that can hybridize to each of the SMB probes with different degree of mismatches. Altogether, these 15 BCs (Figure S6, Supporting Information, *M* = 5 for each of the *N* = 3 fluorescence channels) yielded distinct Tm profiles that may be exploited to theoretically obtain *M*^*N* = 5^3 = 125 unique Tm signatures according to the third‐generation encoding scheme. Video S1 (Supporting Information) shows an example of the image sequences taken during melt‐curve analysis (FAM and HEX channels shown) of this scheme and the corresponding data processing to determine the MET melt curves. For the current setup, the range of melting temperatures that can be detected reliably is from ≈55 °C – 75 °C, and Tm differences of ≈3 °C are easily distinguished. So, we estimate that a multiplexing capability of 6^3^ = 216 can be achieved in a 3‐fluorescence channel equipment. Improvements in instrumentations such as high resolution melting (HRM) capabilities ^[^ ^10b^ ^]^ and additional fluorescence channels could potentially enable even higher degrees of multiplexing. As an experimental proof‐of‐concept, we selected five BCs from the FAM channel, four BCs from HEX channel, and three BCs from ROX channel. Synthetic DNA consisting of flanking universal primer binding sites, and concatenation of one BC from each channel are obtained (5 × 4 × 3 = 60 distinct sequences) (Figure 4B, Data S2, Supporting Information). We performed Tm profiling in three fluorescence channels for each of these sequences separately in the dPCR chip. Unlike the second‐generation scheme where BCs bind to probes in only one channel, probes in all three channels bind to the compound BC in the third‐generation scheme (**Figure** **5**A). We observed that the Tms from individual partitions are highly reproducible (100 positive partitions in dPCR are sampled for each BC in Figure 5A). The positive partitions are randomly distributed across the entire array with no particular bias or edge effects (Figure S7, Supporting Information). Overall, although the different sets of synthetic DNA may share similar BCs in one or two fluorescence channel, the unique Tm combinations from three channels simultaneously allowed unambiguous identification of all 60 sequences. This is also clearly observed in the 3D‐scatter plot of the sequence Tm in dPCR (Figure 5B). Sixty clusters of Tm combinations are clearly distinguishable. A simple classifier, based on a 1 °C × 1 °C × 1 °C cube centered on the median Tms of each sequence, is overall 98.6% sensitive in identifying the compound BC sequence (Table S4, Supporting Information). To our knowledge, the 60‐target identification demonstrated here is the highest degree of multiplexing in dPCR reported to‐date.

Having shown the robust identification of compound BCs with Tm profiling in dPCR, we proceeded to design a third‐generation METEOR‐dPCR assay with expanded multiplexing capability. Based on the 60 compound BCs that we have established, it is possible to assay for 60 targets on a single sample. The other interesting possibility is that by assigning the BCs from one channel to denote different samples instead, METs from multiple samples can be pooled and compared in a single assay. With this pooling, further reduction in the consumables and time to obtain results can be achieved. However, it has to be noted that may also dilute the samples and hence reduce sensitivity. In addition to actual samples, control samples with known copy numbers (e.g., normal diploid DNA) may also be included in each assay (assigned to specific BCs) as a reference to normalize for variations among targets (e.g., different hybridization and ligation efficiencies of METs). With this in mind, we utilized the validated BCs from FAM (5 Tm values) and HEX (4 Tm values) channels to encode for 20 (5 × 4) target genes. The compound BCs of these 20 genes are attached to RHT). Meanwhile, we designed and synthesized three separate pools of LHT that each contained a different BC from ROX channel (3 Tm values) (Data S3, Supporting Information). To each sample, we added the pool of RHTs containing the target gene BCs and a pool of LHTs containing the sample BC. Successfully ligated METs thus contained the gene BC (right), sample BC (left), and flanking universal primer binding sites that enable their PCR amplification.

**Figure 6:**
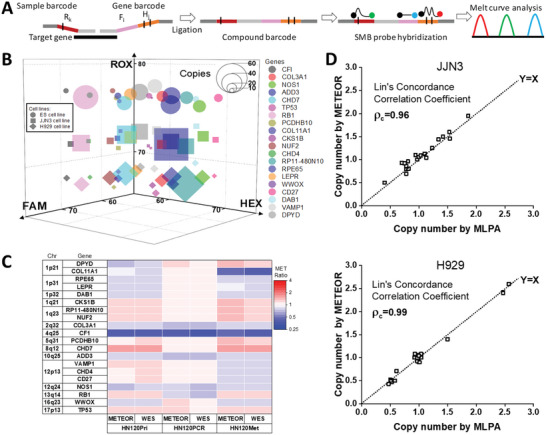
Multiplexed gene copy number analysis in cancer cells using Melt‐Encoded‐Tags for Expanded Optical Readout in digital PCR (METEOR‐dPCR). A) Flow chart for performing METEOR‐dPCR assay using third‐generation scheme. The barcodes (BCs) from FAM (5) and HEX (4) channels encode for 20 target genes, the BCs from ROX (3) channel encode for different samples that can be pooled in a single assay. METs are produced quantitatively by ligation in the presence of target genes. They are then partitioned into the microfluidic dPCR chip for the METEOR‐dPCR assay. B) Absolute copies of METs measured for 20 target genes of genomic DNA from ES, JJN3, and H929 cell lines simultaneously from METEOR‐dPCR assay. C) Comparison between the normalized copy number of 20 target genes in JJN3 and H929 cell line DNA from METEOR‐dPCR assay and reference NGS‐based digital MLPA assay. A number higher than one indicate a gene copy number amplification, while a number less than one indicate a gene copy number deletion. D) High concordance between cancer cell copy number estimated by METEOR‐dPCR and reference digital MLPA assay.

We performed METEOR‐dPCR to simultaneously assess copy number status in 20 target regions of genomic DNA from three cell lines (H1 human embryonic stem cell line (ES), JJN3, and NCI‐H929 (H929)). ES cells have normal diploid genome ^[^ ^24^ ^]^ and the DNA was included as a reference material. Meanwhile, JJN3 and H929 are myeloma cell lines that have been extensively characterized.^[^ ^25^ ^]^ Genomic structural alterations including large‐scale copy number gain and loss are frequently found in myeloma. CNV involving certain genomic loci in multiple myeloma (e.g., del(17p), del(1p32), 1q amplification) are strongly associated with poor outcomes.^[^ ^26^ ^]^ Gene copy number profiling of multiple myeloma is thus becoming an important tool for risk stratification and disease prognosis.^[^ ^27^ ^]^ We plotted the absolute copies of METs detected in dPCR corresponding to Tm profiles of the respective genes. Three groups of measurements, defined by their Tm values in ROX were clearly observed in **Figure** **6**B and Table S5 (Supporting Information). These corresponded to data from three samples that differed in their RHT BCs. Large differences in MET abundance were observed for some target genes between samples, indicative of CNVs. Interestingly, variations in MET abundance is observed even within the reference sample (ES cells). Given that the reference sample is known to have a uniform copy number profile, the variations in MET most likely reflected differences in hybridization and ligation efficiencies of the half‐tags. Similar phenomena have been reported in other ligation‐based assays.^[^ ^28^ ^]^ By normalizing the MET abundance from each sample with the corresponding MET abundance from the reference normal sample, the true copy number can be estimated. The ability to pool actual and reference samples in METEOR‐dPCR for simultaneous gene copy number detection provides an effective means to correct for systematic differences and batch effects.

Figure 6C and Table S6 (Supporting Information) show the normalized copy number of 20 target genes in JJN3 and H929 genomic DNA. The gene copy number of these two cell lines reported previously from MLPA assay was also compared in the same figure.^[^ ^29^ ^]^ The normalized gene copy number obtained from METEOR‐dPCR showed excellent concordance with MLPA assay, validating the accuracy of this rapid approach (Figure 6D). From a disease perspective, JJN3 and H929 contained different genomic features associated with high risk multiple myeloma. TP53 is deleted in JJN3 but has normal copy number in H929. Deletion of 17p, which contains TP53 gene, is one of the most adverse genomic aberration contributing to high risk disease.^[^ ^30^ ^]^ On the other hand, 1q amplification,^[^ ^31^ ^]^ also associated with high risk myeloma, was observed in all three loci in 1q arm in both JJN3 and H929. Interestingly, a markedly higher degree of 1q amplification was found in H929 in both METEOR‐dPCR and MLPA. Some studies have shown that higher copy number gain in 1q is associated with lower OS and PFS (33).^[^ ^32^ ^]^ The quantitative nature of METEOR‐dPCR could allow further prognostication of patients based on the magnitude of copy number changes.

**Figure 7:**
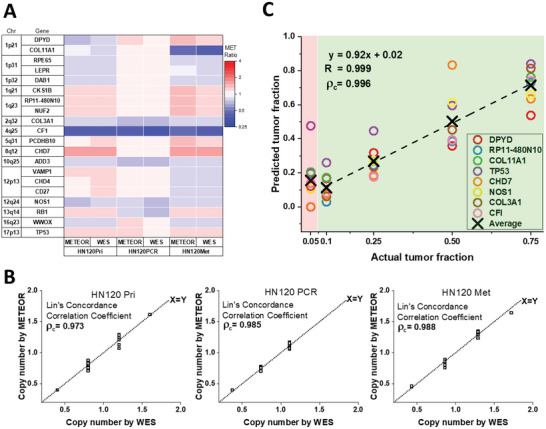
Melt‐Encoded‐Tags for Expanded Optical Readout in digital PCR (METEOR‐dPCR) enables tumor fraction determination. A) METEOR‐dPCR was performed on DNA from patient‐derived head and neck cancer cell lines. The normalized gene copy numbers were compared with gene copy numbers obtained from whole‐exome sequencing of the same cell line for investigating genomic alterations in different stages of cancer. A number higher than one indicate a gene copy number amplification, while a number less than one indicate a gene copy number deletion. B) High concordance between cancer cell copy number estimated by METEOR‐dPCR and reference whole‐exome sequencing (WES) assay. C) METEOR‐dPCR was carried out on samples comprising different proportion of HN120Met DNA mixed with DNA from cells with diploid copy number (ES cell). Multigene analysis enabled by METEOR‐dPCR allowed accurate prediction of tumor fraction down to 10%. Correlation and concordance analysis was performed on tumor fraction measurements from 0.1 to 0.75.

To demonstrate the general applicability of METEOR‐dPCR, we further performed gene copy number profiling in patient‐derived head and neck cancer cell lines. These previously reported cell lines were shown to closely resemble the parental cells in drug vulnerabilities, and are excellent models to model cancer progression.^[^ ^33^ ^]^ To investigate genomic alterations in different stages of cancer, cell lines derived from primary (Pri), metastatic (Met), and drug resistance (PCR) stage of a single patient (HN120) ^[^ ^33^ ^]^ were tested using METEOR‐dPCR for CNV in 20 genes (**Figure** **7**A and Table S7, Supporting Information). To validate the accuracy of METEOR‐dPCR, we also compared our results with copy number profiles inferred from parallel whole‐exome sequencing (WES) of these cells. As before, the normalized gene copy numbers obtained from METEOR‐dPCR showed high concordance with WES, validating its accuracy (Figure 7B). The concurrent gain/loss of copy numbers from genes in the same arm (12p, 1q) indicated that large‐scale copy number changes have occurred in some chromosomes. Comparing between the different cell stage, we observed slight differences in CNV between the metastatic cells and primary cells (e.g., genes in 12p: VAMP1, CD27, CHD4). Interestingly, the drug resistant cells, which were derived by selecting cells that survived long‐term exposure to chemotherapy drugs, had a markedly different compared to both primary and metastatic cells, suggesting major genomic alterations associated with the acquisition of drug resistance phenotype in cancer cells of this patient. Our results showed that apart from detecting CNV between different cancers, METEOR‐dPCR is also capable of deciphering genomic alterations between different stages of cancer of the same patient for understanding the trajectory of cancer progression. Compared to WES, which typically cost above $500,^[^ ^34^ ^]^ METEOR‐dPCR can be performed at much lower cost (≈$75 for 12 arrays in Fluidigm dPCR chip), and thus represents an attractive alternative for targeted genomic profiling in cancer.

Finally, in recent times there is intense interest in profiling of CNV of cell‐free DNA for noninvasive cancer^[^ ^35^ ^]^ or fetal aneuploidy detection.^[^ ^36^ ^]^ In this context, highly sensitive and quantitative methods are required to detect potential copy number alterations against the high background of cfDNA from healthy host cells. Although the use of dPCR has been demonstrated for single gene copy number detection in cfDNA,^[^ ^37^ ^]^ the majority of liquid biopsies applications still relied on next‐generation sequencing due to its improved sensitivity from multiloci profiling. We envision that METEOR‐dPCR could provide enhanced sensitivity to detect subtle change in cfDNA copy numbers due to tumor burden across multiple‐loci compared to conventional single‐target dPCR. To validate this, we mixed different ratios (1:19, 1:9, 1:3, 1:1, 3:1) of cancer DNA (HN120Met) with uniform diploid genomic DNA (ES cell). We performed METEOR‐dPCR on these mixed samples (50 ng µL^−1^ for final concentration) and considered the MET abundance of four loci known to undergo gene amplification (CHD7, TP53, RP11‐480N10, and DYPD) and four loci known to undergo gene loss (CF1, COL3A1, NOS1, and COL11A1) in HN120Met. Based on the previously measured gene copy numbers of normal and cancer cells, we can estimate the tumor fraction of these mixed samples. We observed that while the predicted tumor fraction based on individual genes sometimes deviates from the theoretical expectations, presumably due to sampling uncertainties, average tumor fraction calculations based on all eight loci were highly concordant with the input fractions down to 10% (Figure 7C, Table S8, Supporting Information). This was due to the improved measurement precision when aggregate copy number data from multiple gene loci were used for tumor fraction estimation. This highlights the improvement in quantitative analysis enabled by multigene measurements in METOR‐dPCR, achieving sensitivities comparable to next‐generation sequencing‐based digital MLPA.^[^ ^20^ ^]^ However, we also observed a deviation from linearity of average tumor fraction calculation when the tumor fraction is very low (e.g., 5%). Accurate detection of tumor fractions becomes increasingly challenging when the proportion of cancer DNA is low. Although there could be room for improvement of detection of very low tumor fraction by increasing the number of arrays used in METEOR‐dPCR, this task may be more suited using other high‐throughput methods such as next‐generation deep sequencing that are capable of obtaining millions of measurements. Thus, METEOR‐dPCR could provide a low cost and rapid alternative to next generation deep sequencing for measuring intermediate levels of tumor fractions, such as detection of copy number subclones in tumors or for routine monitoring of cancer patient response to treatment with liquid biopsy.

## 3 Discussion

Genomic CNV are hallmarks of many diseases such as cancer and fetal aneuploidy. With the advent of next generation sequencing, there is great excitement for applying copy number profiling in applications ranging from cancer prognosis and treatment selection, to noninvasive prenatal testing and cancer liquid biopsy. In the majority of these precision medicine applications, highly multiplexed gene copy measurement is required to increase the clinical sensitivity and specificity. Real‐time PCR and dPCR are the most widely used nucleic acid quantification platform in clinical settings due to their ease of use, rapid results and cost‐effectiveness. However, they are currently not compatible with many clinical gene copy number profiling applications due to their limited multiplexing capability.

In this study, we report on the highly multiplexed METEOR‐dPCR platform that can simultaneously quantify 60 targets in a single assay. The novelty of METEOR‐dPCR is based on the integration of three fundamental concepts. Firstly, METs are derived from ligation of probes that hybridize to the target genes. The use of METs as proxies for gene copy number is the key to enabling easy detection (amplification with universal primer) and flexibility to provide unique BCs for multiple targets. Secondly, multicolor melt‐curve analysis significantly expanded the ability to distinguish different MET BCs with the limited fluorescence channels available on existing instruments. With the third‐generation encoding scheme, the maximum multiplexing capacity achievable is *M*^*N*, where *M* is the number of resolvable melting temperatures and *N* is the number of channels. Lastly, complex melt‐curve profiles that arise from mixture of multiple METs are avoided by distributing individual METs in an array of partitions within a dPCR platform. As a result, each partition contains clonally amplified MET that can be unambiguously identified from melt‐curve analysis. Simple enumeration of the partitions with specific melt‐curve profiles will give accurate quantification of the corresponding MET.

There have been other works that demonstrated enhanced multiplexing in bulk PCR or qPCR platforms, including the use melt‐curve analysis^[^ ^38^ ^]^ or amplitude encoding,^[^ ^39^ ^]^ often in instruments that support a large number of fluorescence channels.^[^ ^40^ ^]^ These assays could only qualitatively screen for the presence of certain targets, or quantify a single target. In contrast, METEOR‐dPCR can simultaneously determine the quantity of each target in a mixed sample, making it uniquely suited for multiplexed copy number profiling. We validated the accuracy of METEOR‐dPCR for quantifying CNVs in two different myeloma cell lines JJN3 and H929, and identified genomic aberrations that are known to have prognostic values in multiple myeloma in these samples. We also demonstrated that METEOR‐dPCR is able to track CNV changes during the progression of head‐and‐neck cancer within the same patient. Finally, we showed a proof‐of‐concept of measuring tumor fraction when cancer DNA are mixed with an excess of normal DNA. This highlights the sensitive and quantitative nature of METEOR‐dPCR and its potential uses for liquid biopsy applications.

A conservative estimation of the multiplexing capacity of METEOR‐dPCR, assuming three fluorescence channels and six resolvable Tms per channel, is 216 (6^3^). However, this is not its limits and there are plenty of room for further expansion. Although the standard Fluidigm Biomark HD reader is equipped with three fluorescence channels, it has capacity for five filter sets, bringing the theoretical multiplexing capacity up to 7776 (6^5^). Six color dPCR platforms are also available,^[^ ^41^ ^]^ although these lack melt‐curve measurement capability. A potential issue that may arise with such high degree of multiplexing is the sufficiency of partition numbers in dPCR platforms to accommodate all the METs generated. While the Fluidigm dPCR system has ≈37 k partition, recent innovations in dPCR platforms including the ClarityPlus dPCR system and QuantStudio Absolute Q dPCR system (both ≈320 k partitions), as well as the Qiagen Nanoplate dPCR system (≈816 k partitions per plate) have significantly higher number of partitions that would support further expansion of multiplexing capability through the METEOR‐dPCR principles.^[^ ^42^ ^]^ Meanwhile, improvements in dPCR instrumentation, such as platforms that can perform HRM analysis, could also further augment the capabilities of METEOR‐dPCR to distinguish unique BCs.

Currently METEOR dPCR require real‐time monitoring of fluorescence melt curves, which is possible in commercial platforms that integrates fluorescence detection and thermal. There is no direct way to perform METEOR‐dPCR in end‐point dPCR platforms such as droplet dPCR, but it could be possible to collect droplets after amplification in an imaging chamber with thermal control to observe their fluorescence.^[^ ^43^ ^]^ Finally, there were reports on establishing on‐chip thermal gradients using heaters that can be used to modulate the temperature of droplets as they pass through different zone.^[^ ^44^ ^]^ This could be an interesting way for continuous flow implementation of METEOR‐dPCR on droplet dPCR platform.

In summary, we have demonstrated that METEOR‐dPCR combines the expanded multiplexing capability afforded by melt‐curve analysis with the quantitative aspect of dPCR to enable rapid copy number profiling of gene panels. While this manuscript focuses on DNA copy number analysis, the METEOR‐dPCR principles can potentially be extended to many diverse applications. MLPA have been used for epigenetic studies by quantifying DNA methylation, and gene expression studies by quantifying cDNA.^[^ ^45^ ^]^ Tag ligation is also sensitive to single nucleotide variations and can be used to quantify different SNPs or mutations in DNA samples.^[^ ^46^ ^]^ As all these assays finally generate a ligated tag molecule, it would be straightforward to adapt the METEOR‐dPCR platform for their measurement. With dPCR assays becoming more common in the clinical setting, we envision that METEOR‐dPCR will be a new paradigm for implementing a variety of highly multiplexed multigene assays for rapid testing.

## 4 Experimental Section

### Design of BCs with Variable Affinity to SMB Probes

Each SMB probe consists of a stem region that can self‐hybridize, and a ≈30‐base loop region. BCs with variable affinity to the probe were obtained by designing complementary sequences to the loop region with different numbers and locations of mismatches.^[^ ^14^ ^]^ The DINAMelt software (Two state melting hybridization) ^[^ ^15^ ^]^ was used to estimate the thermodynamic properties of the hybridization between the BCs and SMB probes (parameters: sodium salt at 10 × 10^−3^ m, magnesium salt at 1 × 10^−3^ m, strand concentration at 0.01 × 10^−6^ m). A set of BC sequences with clearly different theoretical Tm were synthesized and tested experimentally to obtain empirical Tm. A set of BC sequences with empirically resolvable Tm are chosen for METEOR‐dPCR experiments.

### Probes and Oligonucleotide Synthesis

All custom oligonucleotide probes and templates were obtained from Integrated DNA Technologies (IDT). Specific fluorophore and quenchers for the SMB probes, as well as 5′‐phsphorylation modification for LHT were added during DNA synthesis. The sequences of all the oligonucleotide probes and templates were listed in Data S1–S3 (Supporting Information).

### Cell Line DNA

Genomic DNA of JJN3 and H929 myeloma cell lines was a kind gift from Wee Joo Chng, Cancer Science Institute of Singapore. Genomic DNA of H1 ES cells was a kind gift from Yi‐Chin Toh, Queensland University of Technology. Genomic DNA of HN120Pri, HN120Met, and HN120PCR was a kind gift from Ramanuj DasGupta, Genome Institute of Singapore.

### Generation of Copy Number Tags (MET)

To perform MET tag hybridization, 1 µL of DNA template, 1 µL of 0.1 × 10^−3^ m LHT, 1 µL of 0.1 × 10^−3^ m RHT, 3.5 µL ddH_2_O, and 1.5 µL SALSA MLPA buffer (MRC Holland) were mixed in a 0.2 mL tube. The mixture was incubated at 95 °C for 5 min and 65 °C for 16 h for the half‐tags to hybridize to the DNA template.

Ligation master‐mix was prepared by mixing 25 µL ddH_2_O, 3 µL ligase buffer A, 3 µL ligase buffer B, and 1 µL Ligase‐65 enzyme (all from MRC Holland) together. To perform MET ligation, the temperature of the hybridization mixture was lowered to 54 °C in the thermocycler, and 32 µL of ligation master‐mix was added into the tube. Ligation reaction was carried out at 54 °C for 15 min. Subsequently, the ligase was inactivated by incubating the reaction at 98 °C for 5 min. The ligation reaction mix was used for the subsequent METEOR‐dPCR analysis without separation of the MET from target DNA molecules. Only the MET would be amplified in dPCR and generate Tm signatures. The DNA targets would not be amplified in dPCR as they do not contain the requisite primer binding sites, so they would not interfere with the final signal.

### Bulk PCR Reaction and Melt‐Curve Analysis

To perform bulk PCR reaction and melt‐curve analysis, 1 µL of ligated MET (above), 1 µL of 0.5 × 10^−3^ m SMB probes, 1.25 µL of 0.5 × 10^−3^ m forward primer, 1.25 µL of 10 × 10^−3^ m reverse primer, 5 µL of TaqMan Fast Advanced Master Mix (Thermo Fisher Scientific), and 0.5 µL ddH_2_O were mixed together. The mixture was sealed and placed in a qPCR machine (Biorad CFX96 Touch Real‐Time PCR Detection System). Asymmetric PCR was performed with the following thermal conditions: 30 cycles of 95 °C 10 s; 56 °C 20 s; 70 °C 30 s. To perform melt‐curve analysis, the reaction mixture was first incubated at 95 °C for 5 min and then cooled down to 40 °C for 5 min. The reaction mixture was heated gradually from 40 °C to 95 °C (0.5 °C interval, soak time 10 s), with continuous monitoring of fluorescence during the entire process for melt‐curve analysis.

### METEOR‐dPCR Assay

The Digital PCR 37K IFC (Fluidigm) was utilized that contained 48 × 770 compartments (0.85 nL each compartment) to perform METEOR‐dPCR assay. The reaction mixture for each METEOR‐dPCR assay consisted of the following: 0.5 µL of template DNA (ligated MET or synthetic DNA BCs), 1 µL of 0.5 × 10^−3^ m SMB probes, 0.5 µL of 0.5 × 10^−3^ m forward primer, 0.5 µL of 10 × 10^−3^ m reverse primer, 2.5 µL of TaqMan Fast Advanced Master Mix (Thermo Fisher Scientific), and 0.25 µL 20 × GE Sample Loading Reagent (Fluidigm).

A total of 5 µL of sample was loaded into each sample inlet and 10 µL of 1X GE Sample Loading Reagent was loaded into all the hydration inlets. The Fluidigm IFC Controller MX was used to prime and load the dPCR chip. After chip priming and sample loading, dPCR was performed according to the manufacturer's instructions in the Fluidigm Biomark HD system using the following thermal conditions: 60 cycles of 95 °C 10 s; 56 °C 20 s; 70 °C 30 s. To perform melt‐curve analysis, the reaction mixture was first incubated at 95 °C for 3 min and then cooled down to 40 °C for 5 min. The reaction mixture was heated gradually from 44 °C to 88 °C (1 °C interval), with continuous monitoring of fluorescence signal (FAM, HEX, ROX) during the entire process for melt‐curve analysis. The Fluidigm Digital PCR Analysis Program (version 4.1.2) was used to obtain the melting temperature in each partition. The Tm values were exported into Microsoft Excel for subsequent analysis. The number of partitions with Tm values within ±0.5 °C of the median Tm of each BC (determined previously from METEOR‐dPCR of synthetic BC) were summarized for subsequent copy number analysis.

### Normalization to Determine Genomic Copy Number

To determine the relative copy number of target genes, the normalization approach employed was adapted in MLPA assays. A reference sample known to have normal copy numbers (e.g., ES cell DNA) was profiled with METEOR‐dPCR in parallel with the actual samples. The relative copy number of each gene in the sample was calculated as (Number of partitions with specific BC Tm in sample)/(Number of partitions with specific BC Tm in reference).

### Tumor Fraction Estimation in Mixture Experiments

The genomic copy number of each target gene in 100% tumor DNA (CP_tumor_) was predetermined with METEOR‐dPCR. To perform the mixture experiment, different ratios of tumor DNA and normal DNA (ES cell DNA) were combined. The genomic copy number of each target gene in the sample (CP_sample_) was obtained through METEOR‐dPCR experiment and normalized by reference sample as described above. The tumor fraction α of the sample is estimated by considering the following equation: CP_sample_ = *α* CP_tumor_ + (1 − *α*) CP_ref_. As the copy number of the reference is 1, rearrangement of this equation gives:

(1) $$\alpha =\frac{{\mathrm{CP}}_{\mathrm{sample}}\;-\;1}{{\mathrm{CP}}_{\mathrm{tumor}}\;-\;1}$$

### Whole Exome Sequencing (WES)

The Agilent SureSelect V6 58 Mb kit was used to prepare sequencing libraries from HN120Pri, HN120Met and HN120PCR genomic DNA. Samples were sequenced on an Illumina NovaSeq PE150 platform at 12 GB data/100X exome coverage. Raw WES data were processed through GATK best practices pipeline, and copy number segmentation and calling were done with CNVKit.

## Conflict of Interest

The authors declare no conflict of interest.

## Supporting information

Supporting Information

Supplemental Video 1

Supplemental Table 1

## Data Availability

The data that support the findings of this study are available in the supplementary material of this article.
