# Supplementary material for: Melt‐Encoded‐Tags for Expanded Optical Readout in Digital PCR (METEOR‐dPCR) Enables Highly Multiplexed Quantitative Gene Panel Profiling
Source: Adv Sci (Weinh). 2023 Jul 23;10(27):2301630. doi: 10.1002/advs.202301630 (PMC10520687; doi:10.1002/advs.202301630)
Supplement: Supplementary file 1 — Supporting Information [file ADVS-10-2301630-s002.pdf]

ADVANCED SCIENCE
Open Access

Supporting Information

for *Adv. Sci.*, DOI 10.1002/advs.202301630

Melt-Encoded-Tags for Expanded Optical Readout in Digital PCR (METEOR-dPCR) Enables Highly Multiplexed Quantitative Gene Panel Profiling

*Dong Dong Liu, Daniel Muliaditan, Ramya Viswanathan, Xu Cui and Lih Feng Cheow**

Supporting Information for

Melt-Encoded-Tags for Expanded Optical Readout in digital PCR (METEOR-dPCR) enables highly multiplexed quantitative gene panel profiling

Dongdong Liu, Daniel Muliaditan, Ramya Viswanathan, Xu Cui, Lih Feng Cheow

Corresponding author – Lih Feng Cheow
Email: bieclf@nus.edu.sg

This PDF file includes:

Figures S1 to S7
Tables S1 to S8
Legends for Supplementary Data S1 to S3

Other supporting materials for this manuscript include the following:

Supplementary Data S1 to S3
Supplementary Video 1

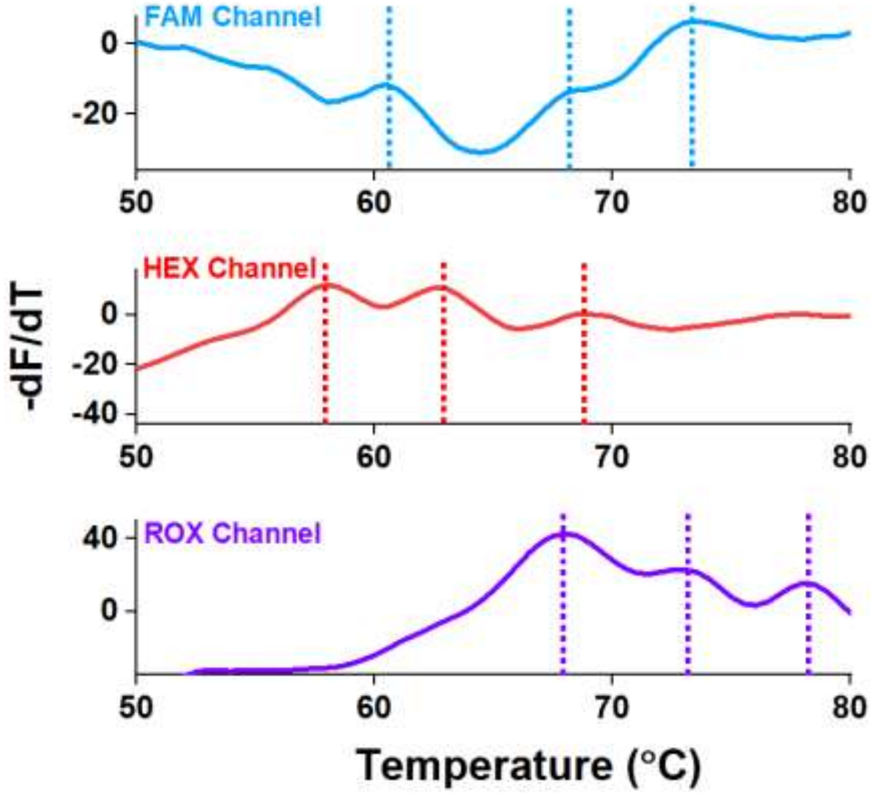

Fig. S1. Multiple melt curve peaks in real-time PCR corresponding to all nine METs were detected when genomic DNA of JJN3 cells was tested. 1 μL of 100 ng/μL JJN3 cell DNA as used as template. Probe mix for 9 genes (second-generation scheme) was added for ligation reaction.

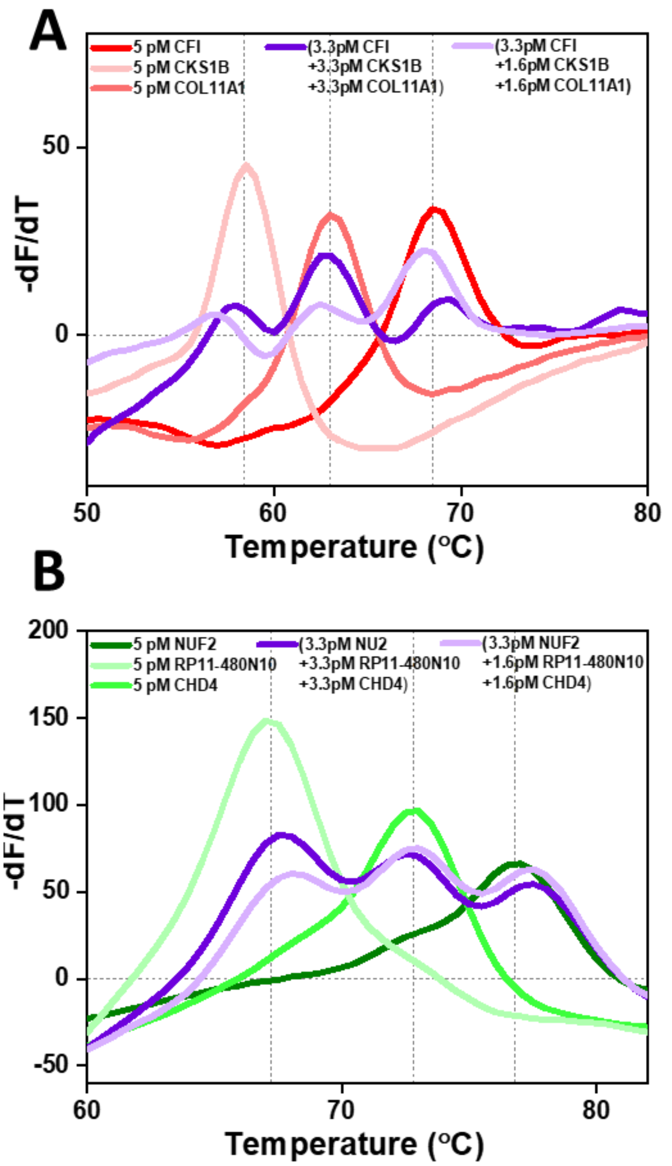

Fig. S2. Single and multiple target METs detected via melt curve analysis after asymmetric PCR in real-time PCR platform. 1 μL of synthetic DNA template with the indicated concentration was used as target. Probe mix for 9 genes (second-generation scheme) was added for ligation reaction. Shown are melt curve profiles in HEX channel (A) and ROX channel (B).

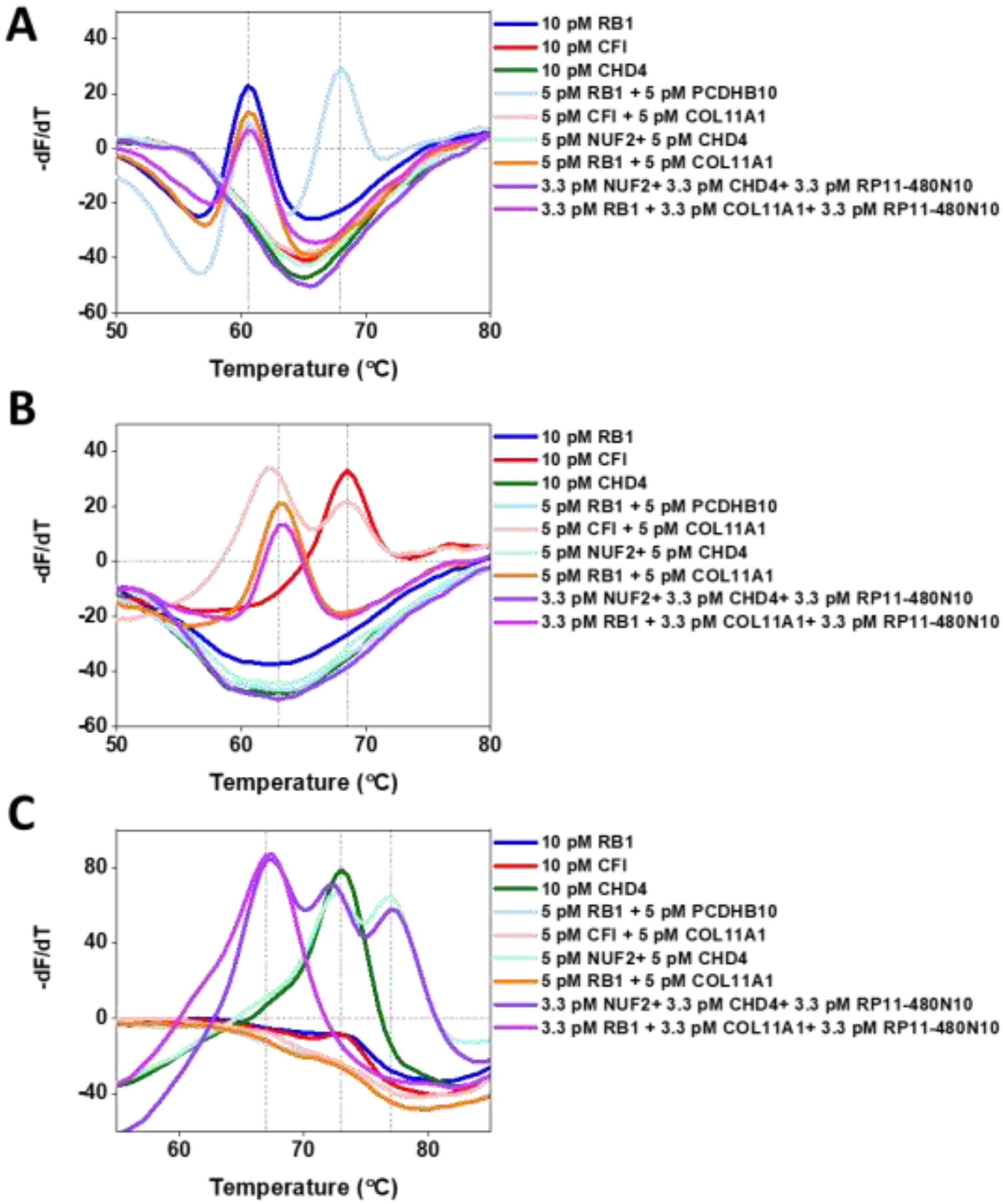

Fig. S3. Single and multiple target METs detected via melt curve analysis after asymmetric PCR in real-time PCR platform. 1 μL of synthetic DNA template with the indicated concentration was used as target. Probe mix for 9 genes (second-generation scheme) was added for ligation reaction. Shown are melt curve profiles in FAM channel (A), HEX channel (B) and ROX channel (C)

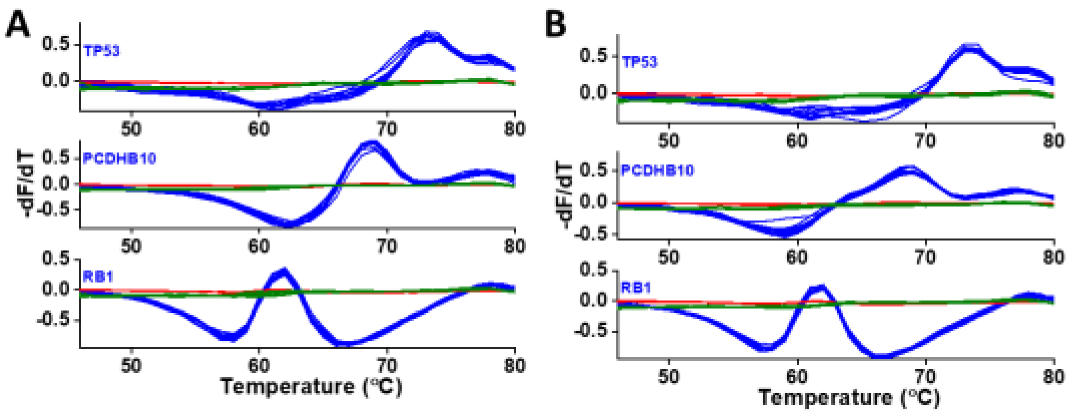

Fig. S4. Additional melt curve profiles in ten nanoliter partitions from two other dPCR chips reflecting the reproducibility of METEOR-dPCR assay.

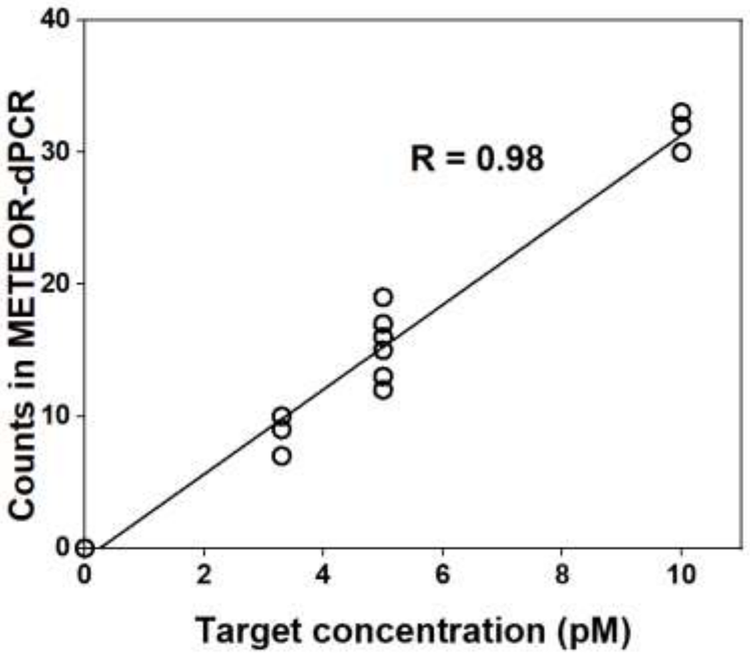

Fig. S5. Linear relationship between the number of positive partitions (with expected Tm) in METEOR-dPCR and input target gene concentrations.

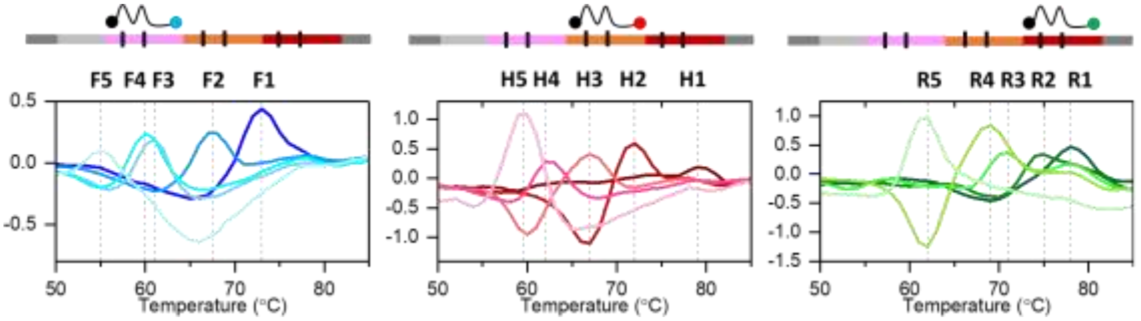

Fig. S6. Demonstration that 5 distinct Tm's can be obtained in each fluorescence channel with the appropriate barcodes. According to the third-generation encoding scheme, this would theoretically allow 125 unique compound Tm signatures (5^3) to be utilized.

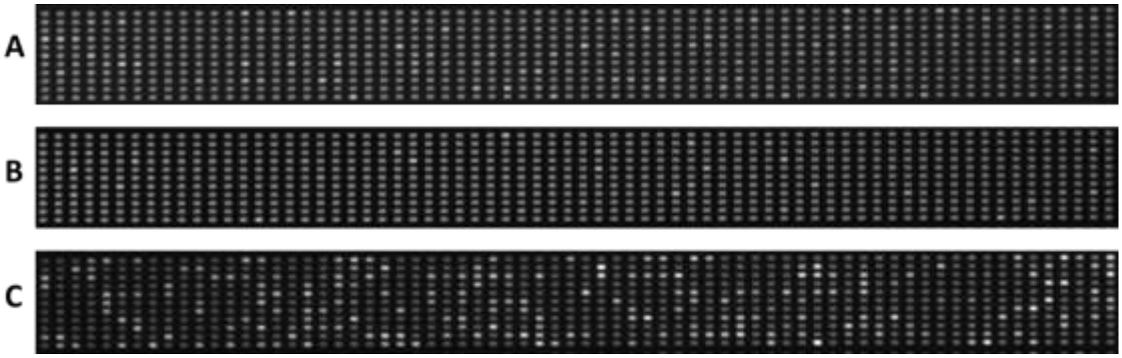

Fig. S7. Characteristic fluorescence images for METEOR-dPCR from FAM (A), HEX (B) and ROX (C) channels.

Table S1. Observed Tm values of the nine METs designed according to the second-generation scheme in METEOR-dPCR. The median Tm and standard deviation (from 50 measurements) are displayed.

MET / Gene ID	FAM Tm (°C)	HEX Tm (°C)	ROX Tm (°C)
CFI	-	68.3±0.1	-
COL11A1	-	62.6±0.1	-
CKS1B	-	57.9±0.1	-
NUF2	-	-	76.8±0.6
CHD4	-	-	73.3±0.3
RP11-480N10	-	-	67.9±0.2
TP53	75.5±0.3	-	-
RB1	61.4±0.2	-	-
PCDHB10	68.1±0.2	-	-

Table S2. Quantification of MET corresponding to different input concentrations of single synthetic DNA template in METEOR-dPCR according to second-generation scheme.

**Sample**	**Number of Partition**	**Sample**	**Number of Partition**	**Sample**	**Number of Partition**
NTC	0	NTC	0	NTC	0
5 pM RB1	9	5 pM CFI	9	5 pM RP11-480N10	10
10 pM RB1	18	10 pM CFI	19	10 pM RP11-480N10	19
20 pM RB1	38	20 pM CFI	35	20 pM RP11-480N10	41
40 pM RB1	75	40 pM CFI	69	40 pM RP11-480N10	74
80 pM RB1	146	80 pM CFI	142	80 pM RP11-480N10	154

Table S3. Quantification of MET corresponding to mixtures of different synthetic DNA template in METEOR-dPCR according to second-generation scheme. There are three repetitions for each group.

	Partitions with corresponding Tm values								
**Sample**	**RB1**	**PCDHB10**	**TP53**	**CKS1B**	**COL11A1**	**CFI**	**RP11-480N10**	**CHD4**	**NUF2**
**NTC**	0	0	0	0	0	0	0	0	0
	0	0	0	0	0	0	0	0	0
	0	0	0	0	0	0	0	0	0
**10 pM RB1**	**32**	0	0	0	0	0	0	0	0
	**28**	0	0	0	0	0	0	0	0
	**27**	0	0	0	0	0	0	0	0
**10 pM CFI**	0	0	0	0	0	**30**	0	0	0
	0	0	0	0	0	**27**	0	0	0
	0	0	0	0	0	**29**	0	0	0
**10 pM CHD4**	0	0	0	0	0	0	0	**33**	0
	0	0	0	0	0	0	0	**28**	0
	0	0	0	0	0	0	0	**27**	0
**5 pM RB1 + 5 pM PCDHB10**	**19**	**13**	0	0	0	0	0	0	0
	**17**	**19**	0	0	0	0	0	0	0
	**18**	**12**	0	0	0	0	0	0	0
**5 pM CFI + 5 pM COL11A1**	0	0	0	0	**15**	**12**	0	0	0
	0	0	0	0	**19**	**11**	0	0	0
	0	0	0	0	**17**	**12**	0	0	0
**5 pM NUF2+ 5 pM CHD4**	0	0	0	0	0	0	0	**15**	**15**
	0	0	0	0	0	0	0	**11**	**18**
	0	0	0	0	0	0	0	**19**	**13**
**5 pM RB1 + 5 pM COL11A1**	**16**	0	0	0	**17**	0	0	0	0
	**16**	0	0	0	**15**	0	0	0	0
	**17**	0	0	0	**14**	0	0	0	0
**3.3 pM NUF2+ 3.3 pM CHD4+ 3.3 pM RP11-480N10**	0	0	0	0	0	0	**9**	**10**	**10**
	0	0	0	0	0	0	**9**	**13**	**11**
	0	0	0	0	0	0	**8**	**11**	**9**
**3.3 pM RB1 + 3.3 pM COL11A1+ 3.3 pM RP11-480N10**	**7**	0	0	0	**9**	0	**10**	0	0
	**7**	0	0	0	**11**	0	**9**	0	0
	**11**	0	0	0	**8**	0	**10**	0	0

Table S4. Median Tm's of synthetic compound barcodes (100 each) measured in METEOR-dPCR. Sensitivity denotes the percentage of partitions that fall within ±0.5 °C of the median Tm in each fluorescence channel.

Compound Barcode			Median Tm (°C)			Sensitivity
**F**	**H**	**R**	**F**	**H**	**R**	%
1	1	1	73.7	72.0	77.7	100
1	1	2	74.0	72.4	73.1	100
1	1	3	73.9	72.0	69.2	100
1	2	1	73.9	67.8	77.3	100
1	2	2	73.1	67.0	73.1	100
1	2	3	73.1	67.1	70.1	100
1	3	1	73.9	60.0	77.8	100
1	3	2	73.7	60.3	72.2	100
1	3	3	73.8	59.6	69.6	100
1	4	1	72.7	58.3	77.0	100
1	4	2	73.0	58.2	71.1	100
1	4	3	73.0	58.2	68.0	100
2	1	1	68.5	70.9	78.2	97
2	1	2	68.3	70.8	73.1	100
2	1	3	68.7	70.9	69.6	100
2	2	1	68.6	68.7	78.6	100
2	2	2	68.9	67.8	72.1	100
2	2	3	69.0	67.9	68.6	100
2	3	1	69.3	60.7	77.4	100
2	3	2	69.1	60.8	72.0	100
2	3	3	69.1	60.6	68.4	100
2	4	1	66.8	58.5	75.7	100
2	4	2	68.2	59.2	71.0	94
2	4	3	67.2	58.7	66.6	96
3	1	1	63.9	70.5	77.2	100
3	1	2	63.9	70.7	71.2	100
3	1	3	63.9	70.3	67.8	100
3	2	1	64.1	67.0	78.2	100
3	2	2	64.3	67.2	72.8	100
3	2	3	64.2	66.9	69.7	92
3	3	1	64.9	61.0	77.7	68
3	3	2	64.7	60.1	72.9	100
3	3	3	64.8	60.2	69.7	100
3	4	1	63.2	58.9	76.3	100
3	4	2	63.6	59.2	71.6	100
3	4	3	62.8	58.4	69.8	100
4	1	1	60.7	70.3	76.8	100
4	1	2	60.8	70.5	71.6	100
4	1	3	60.9	70.2	67.8	100
4	2	1	60.7	66.2	77.2	100
4	2	2	60.7	66.3	71.6	100
4	2	3	60.9	66.6	66.9	100
4	3	1	61.2	60.4	78.2	100
4	3	2	61.1	60.2	72.6	100
4	3	3	60.9	60.6	70.1	100
4	4	1	61.2	58.0	77.8	100
4	4	2	61.0	58.1	72.3	100
4	4	3	61.0	56.8	69.1	100
5	1	1	55.9	71.3	78.2	100
5	1	2	55.8	71.3	72.8	100
5	1	3	55.4	70.6	67.8	99
5	2	1	55.9	67.1	78.2	100
5	2	2	55.8	67.0	73.0	100
5	2	3	55.7	67.0	69.5	96
5	3	1	55.8	60.7	78.6	100
5	3	2	55.8	60.8	73.1	100
5	3	3	55.8	60.8	69.9	98
5	4	1	55.2	56.8	76.7	78
5	4	2	55.3	57.1	71.7	98
5	4	3	53.2	57.3	69.3	100

*Overall sensitivity = 98.6%

Table S5. Number of partitions with the characteristic Tm corresponding to the compound barcode of each gene target / cell for copy number analysis in METEOR-dPCR (third-generation scheme). 12 arrays are used.

Gene	Cells	Compound barcode			Partitions
		F	H	R	
TP53	ES	1	1	1	12
	JJN3	1	1	2	6
	H929	1	1	3	13
PCDHB10	ES	1	2	1	10
	JJN3	1	2	2	11
	H929	1	2	3	9
RB1	ES	1	3	1	56
	JJN3	1	3	2	44
	H929	1	3	3	29
CFI	ES	1	4	1	12
	JJN3	1	4	2	15
	H929	1	4	3	13
COL11A1	ES	2	1	1	42
	JJN3	2	1	2	82
	H929	2	1	3	21
CKS1B	ES	2	2	1	5
	JJN3	2	2	2	8
	H929	2	2	3	13
NUF2	ES	2	3	1	12
	JJN3	2	3	2	18
	H929	2	3	3	29
CHD4	ES	2	4	1	14
	JJN3	2	4	2	12
	H929	2	4	3	10
RP11-480N10	ES	3	1	1	29
	JJN3	3	1	2	42
	H929	3	1	3	69
COL3A1	ES	3	2	1	26
	JJN3	3	2	2	24
	H929	3	2	3	27

Gene	Cells	Compound barcode			Partitions
		F	H	R	
CHD7	ES	3	3	1	35
	JJN3	3	3	2	51
	H929	3	3	3	49
ADD3	ES	3	4	1	16
	JJN3	3	4	2	15
	H929	3	4	3	17
NOS1	ES	4	1	1	29
	JJN3	4	1	2	28
	H929	4	1	3	29
RPE65	ES	4	2	1	41
	JJN3	4	2	2	43
	H929	4	2	3	38
LEPR	ES	4	3	1	30
	JJN3	4	3	2	34
	H929	4	3	3	27
WWOX	ES	4	4	1	11
	JJN3	4	4	2	11
	H929	4	4	3	11
CD27	ES	5	1	1	20
	JJN3	5	1	2	16
	H929	5	1	3	10
DAB1	ES	5	2	1	15
	JJN3	5	2	2	16
	H929	5	2	3	14
VAMP1	ES	5	3	1	36
	JJN3	5	3	2	25
	H929	5	3	3	17
DPYD	ES	5	4	1	44
	JJN3	5	4	2	48
	H929	5	4	3	19

Table S6. Comparison of measured gene copy number in JJN3 and H929 genomic DNA between METEOR-dPCR assay and previously reported MLPA assay (MLPA).

		JJN3		H929	
**Chr**	**Gene**	**METEOR**	**MLPA**	**METEOR**	**MLPA**
17p13	TP53	0.5	0.4	1.08	1.04
16q23	WWOX	1	0.97	1	0.92
13q14	RB1	0.79	0.78	0.52	0.5
12q24	NOS1	0.96	0.81	1	1.04
12p13	CHD4	0.86	0.78	0.71	0.61
12p13	CD27	0.8	0.82	0.5	0.58
12p13	VAMP1	0.69	0.78	0.47	0.53
10q25	ADD3	0.93	0.7	1.06	0.97
8q12	CHD7	1.46	1.54	1.4	1.5
5q31	PCDHB10	1.1	0.94	0.9	1
4q25	CFI	1.25	1.23	1.08	0.93
2q32	COL3A1	0.92	0.75	1.04	1
1q23	NUF2	1.5	1.42	2.42	2.47
1q23	RP11-480N10	1.44	1.34	2.4	2.47
1q21	CKS1B	1.6	1.53	2.6	2.57
1p32	DAB1	1.06	1	0.93	0.99
1p31	RPE65	1.04	1.13	0.92	0.97
1p31	LEPR	1.13	1.1	0.9	1.01
1p21	COL11A1	1.95	1.86	0.5	0.51
1p21	DPYD	1.09	1.03	0.43	0.47

Table S7. Comparison of gene copy number in patient-derived head and neck cancer cell lines between METEOR-dPCR assay and whole-exome sequencing (WES).

		**HN120Pri**		**HN120PCR**		**HN120Met**	
**Chr**	**Gene**	**METEOR**	**WES**	**METEOR**	**WES**	**METEOR**	**WES**
17p13	TP53	1.25756	1.2	1.16788	1.11	1.29056	1.29
16q23	WWOX	0.80613	0.8	1.15278	1.11	0.75849	0.86
13q14	RB1	1.2495	1.2	0.78027	0.74	1.23396	1.29
12q24	NOS1	0.70939	0.8	0.76516	0.74	0.80377	0.86
12p13	CD27	1.12858	1.2	1.11754	1.11	0.75849	0.86
12p13	CHD4	1.07215	1.2	1.13265	1.11	0.84906	0.86
12p13	VAMP1	1.2898	1.2	1.05714	1.11	0.84906	0.86
10q25	ADD3	0.80613	0.8	0.7551	0.74	0.84906	0.86
8q12	CHD7	1.61225	1.6	1.06217	1.11	1.64151	1.72
5q31	PCDHB10	1.07215	1.2	1.13265	1.11	1.32453	1.29
4q25	CF1	0.40306	0.4	0.40272	0.37	0.45283	0.43
2q32	COL3A1	0.76582	0.8	0.70476	0.74	0.83773	0.86
1q23	NUF2	1.20919	1.2	1.09237	1.11	1.32453	1.29
1q23	RP11-480N10	1.28174	1.2	1.07727	1.11	1.34717	1.29
1q21	CKS1B	1.20919	1.2	1.13265	1.11	1.32079	1.29
1p32	DAB1	0.80613	0.8	1.09237	1.11	0.80377	0.86
1p31	LEPR	0.85449	0.8	1.0672	1.11	0.89434	0.86
1p31	RPE65	0.83031	0.8	1.08231	1.11	0.86038	0.86
1p21	COL11A1	0.87868	0.8	1.07224	1.11	0.46415	0.43
1p21	DPYD	0.75776	0.8	1.15278	1.11	1.33585	1.29

Table S8. Tumor fraction (TF) estimation in mixed samples based on METEOR-dPCR assay (tumor DNA mixed with ES cell DNA)

**Samples**	**0.05 TF**	**0.10 TF**	**0.25 TF**	**0.50 TF**	**0.75 TF**
**Gene**	**Predicted TF by METEOR-dPCR**				
DPYD	0	0.16667	0.31744	0.35842	0.53763
RP11-480N10	0.19678	0.02924	0.24474	0.38511	0.73099
COL11A1	0.20456	0.17236	0.26903	0.39346	0.76271
TP53	0.47619	0.25974	0.44643	0.59524	0.84034
CHD7	0	0.07286	0.18518	0.83333	0.63492
NOS1	0.10610	0.05747	0.27207	0.61408	0.65866
COL3A1	0.12171	0.06138	0.23546	0.45249	0.81361
CFI	0.13889	0.08333	0.17544	0.39216	0.74074
**Average**	0.15553	0.11288	0.26822	0.50304	0.71495

Data S1 (separate file). (Supplementary Data.xlsx)

Probes and oligonucleotide sequences for METEOR-dPCR assay using the second-generation scheme.

Data S2 (separate file). (Supplementary Data.xlsx)

oligo sequences and their corresponding characteristic Tm values on each channel.

Data S3 (separate file). (Supplementary Data.xlsx)

Probes and oligonucleotide sequences for METEOR-dPCR assay using the third-generation scheme.

Supplementary Video 1(separate file).

An example of the image sequences taken during melt curve analysis (FAM and HEX channels shown) of METEOR-dPCR and the corresponding data processing to determine the MET melt curves.
